# Metastatic involvement of the spleen by endometrial adenocarcioma; a rare asylum for a common malignancy: a case report

**DOI:** 10.1186/1756-0500-6-476

**Published:** 2013-11-19

**Authors:** Adnan Arif, Zain Ul Abideen, Naeem Zia, Muhammad Atif Khan, Tariq Nawaz, Asif Zafar Malik

**Affiliations:** 1 Surgical Unit 2, Holy Family Hospital, Satellite town, Rawalpindi, Pakistan; 2 Department of Anesthesia, Pain and Intensive care, Holy Family Hospital, Rawalpindi Medical College, Rawalpindi, Pakistan; 3 Surgical Unit 2, Holy Family Hospital , Rawalpindi Medical College, Rawalpindi, Pakistan

**Keywords:** Splenic metastasis, Endometrial adenocarcinoma, Splenectomy, Computerized tomography, Ultrasonography

## Abstract

**Background:**

Metastatic involvement of the spleen by solid tumors is a rare clinical entity; those coming from endometrial adenocarcinomas are exceptionally rare. Spleen is an uncommon site for metastatic deposits due to its specific anatomy and microenvironment. Typically, splenic metastasis from endometrial carcinomas present months to years after curative surgery, chemotherapy or radiotherapy. The most common complaint in symptomatic patients is abdominal pain localized to the left hypochondrium. Most however, are asymptomatic only to be picked up on vigilant routine ultrasonography or computerized tomography during follow up. We report the case of a 54-year-old woman who presented to us after 50 months of total abdominal hysterectomy and bilateral salpingo-oophorectomy for an endometrial adenocarcinoma. She had severe abdominal pain localized to the left hypochondrium as the presenting complaint. To the best of our knowledge, this is the 1^st^ case to be reported from Pakistan with 14 cases reported prior to our report. All past cases report the endometroid variant of endometrial adenocarcinoma as the primary tumor and our patient was a victim to the same variant.

**Case presentation:**

A 54-year-old, nulliparous widowed woman presented with severe abdominal pain in the left hypochondrium for the last 4 months. The pain radiated to the left shoulder and was exacerbated with deep breathing. She had a history of total abdominal hysterectomy with bilateral salpingo-oophorectomy done 50 months back for stage 1a endometroid endometrial adenocarcinoma. Clinical examination revealed tenderness in the left hypochondrium but no visceromeglay was appreciable. Ultrasonography and computerized tomography revealed a space-occupying lesion within the spleen with associated splenomegaly. Computed tomography further suggested a large splenic abscess however the patient did not have fever, vomiting or leukocytosis which are the hallmarks of a splenic abscess. A splenectomy was performed for her complaints. On histopathology a metastatic adenocarcinoma was identified consistent with the primary tumor. The tumor was CK7, CA-125 and epithelial membrane antigen positive (EMA). The patient was then referred for further chemotherapy.

**Conclusion:**

From this case we conclude, that although very rare, the spleen is a potential site for metastasis in endometroid endometrial adenocarcinoma. Since most patients are asymptomatic, routine examinations and imaging can identify its presence and avoid complications. If the practice is employed with vigilance, we may expect the clinical event to be diagnosed more frequently. The standard treatment is a classic splenectomy followed by chemotherapy.

## Background

The spleen is a peculiar site for metastasis from solid tumors; most commonly though, they are from breast, lung, colorectal cancers and melanomas. The most cost common causes of solitary or isolated splenic metastasis are colorectal and ovarian cancers. These deposits are usually located on the splenic capsule and signify disseminated disease with a grim prognosis [1]. Splenic metastasis from endometrial carcinoma is considered exceptional; to date, only 14 cases have been reported in the literature and this is the 1^st^ case to be reported from Pakistan. These deposits are always solitary in the spleen, invade the parenchyma only and metastasize via the haematogenous route. However, they do indicate a better prognosis [2]. They usually present after a characteristic latent period; months to years after curative surgery for the initial tumor. They may present with painful splenomegaly or may be completely asymptomatic only to be discovered on relevant imaging during follow up. The cure accepted worldwide is a classic splenectomy followed by post operative chemotherapy. This case is accompanied by a brief review of the literature to highlight important clinical aspects of the disease.

### Case presentation

**Figure 1:**
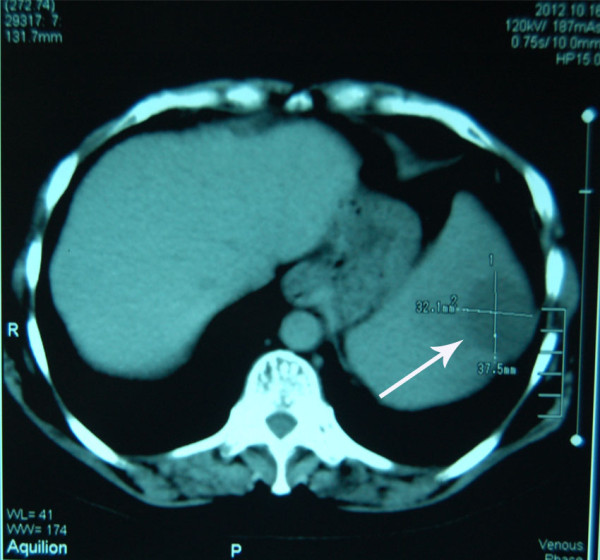
Abdominal computed tomography showing the space occupying lesion inside the spleen (arrow).

**Figure 2:**
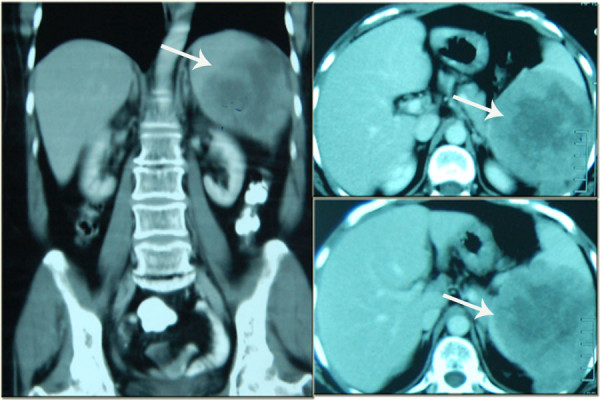
Various views of the abdominal computed tomography showing the location of the space occupying lesion in the spleen (arrow).

A 54-year-old, nulliparous and widowed house wife presented 4 years ago with post menopausal bleeding for 2 months duration. She underwent clinical evaluation and was diagnosed as a case of endometroid adenocarcinoma of the endometrium; stage 1a and moderately well differentiated. She underwent a total abdominal hysterectomy and bilateral salpingo-oophorectomy. Histopathology confirmed the diagnosis, peritoneal washings for malignant cells were negative and computed tomography (CT) scan did not reveal any extra uterine spread of the tumor. Histopathology of the ovaries and fallopian tubes was unremarkable as well. Fifty months later she presented with severe abdominal pain in the left hypochondrium radiating to the tip of the left shoulder for 4 months. The pain was described as dull and dragging, aggravated by deep breathing and occasionally relieved by analgesics; during the last 2 weeks she had multiple acute flare ups. On examination her abdomen was tender in the left hypochondrium without any palpable visceromegaly. Ultrasonography showed an enlarged spleen with a space occupying lesion having well defined walls in its lower part. This was clarified by CT (Figures 1 and 2); the spleen measured 12×9×9 cm with a splenic index of 931. A large heterogeneous well defined lesion was seen in the spleen with peripheral enhancing and central necrosis. It measured about 9×9×8 cm. A small exophytic component was also seen across the left posterolateral wall. There was peri splenic fat stranding with thickening of the Zukerkandl and lateral coronal fascia. An opinion of splenic abscess was given by the radiologist however the patient did not have the classic signs of fever, vomiting and leukocytosis. All other investigations including echocardiograms, blood and urine cultures were normal and no definite focus for the suspected splenic abscess was isolated.

**Figure 3:**
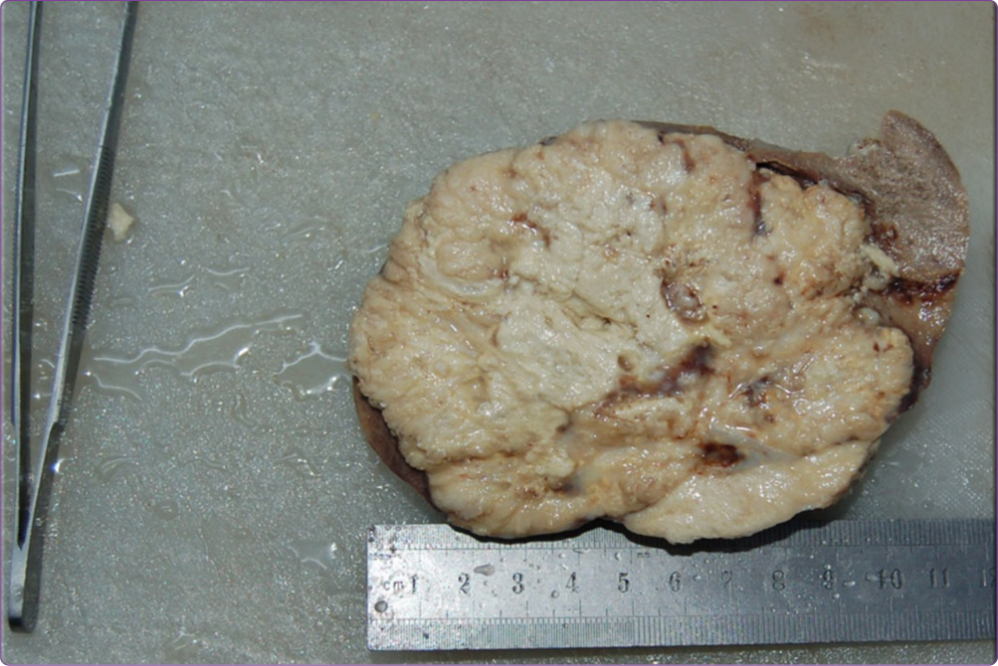
**Gross specimen of the spleen after splenectomy.** A mass can be easily appreciated in the centre of the spleen extending to the margins of the specimen; there is diffuse infiltration of the parenchyma.

**Figure 4:**
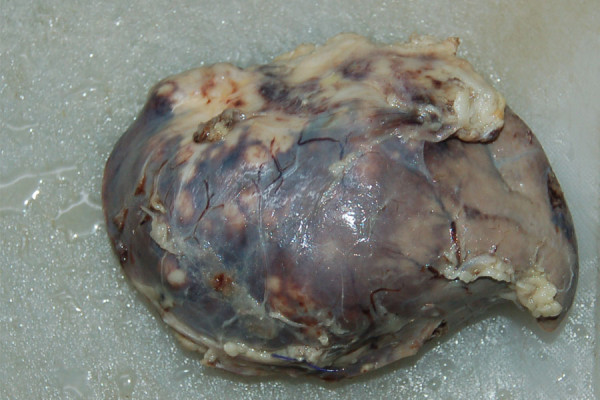
Another view of the gross specimen clearly showing no involvement of the splenic capsule which is characteristic for endometroid endometrial adenocarcimona involvement of the spleen.

To alleviate patient symptoms a classic elective splenectomy was performed. During the operation multiple adhesions of the spleen with surrounding areas were noted and an enlarged spleen containing a central necrotic zone was extracted as shown in Figure 3. Figure 4 shows the histopathology of the gross specimen revealing a metastatic adenocarcinoma. Search for the primary in the gastrointestinal tract proved inconclusive. The tumor was CA-125, CK-7 and epithelial membrane antigen (EMA) positive. Vimentin, CK20, carcinoembryonic antigen (CEA) and CDX2 were all negative. The patient was discharged and referred for post-op chemotherapy. Her pain was relieved and she felt much better on discharge.

## Discussion

Solitary splenic metastasis is very uncommon and close to 100 such cases have been reported in the literature while those originating from endometrial adenocarcinoma are considered exceptional. Half of these are from the female genital tract; most commonly from ovarian malignancies (more than 30 cases) and the remainder from endometrial (13 cases), cervical (6 cases) and tubal carcinomas (1 case) [2].

Splenic metastasis are most commonly incidentally detected by ultrasound (US) or CT during follow up or work up of patients with cancer. When solitary, about 60% are asymptomatic. Prominent symptoms at presentation include fatigue, weight loss, fever, and/or abdominal pain while signs include splenomegaly with associated anemia or thrombocytopenia while rarely presentation may be with splenic rupture [2]. Occasionally solitary splenic metastasis may be the first manifestation of recurrent solid cancers in particular gynecologic malignancies [3]. A review had shown 33% of solitary splenic metastasis were discovered on routine examination while they were completely asymptomatic [1]. Our patient only had a history of dull abdominal pain which gradually increased in severity which prompted the patient to seek emergency care.

**Figure 5:**
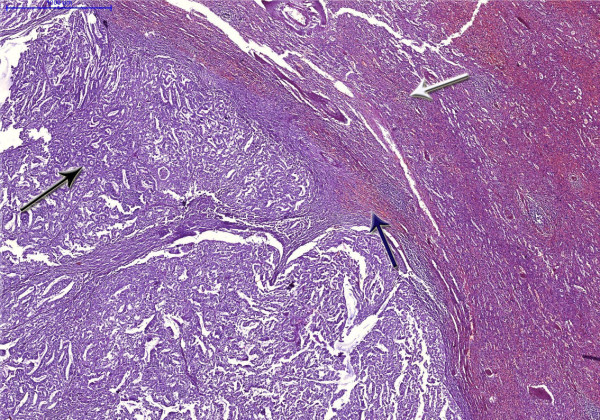
**Histopathology of the specimen.** The black arrow shows the endometrial tissue comprising neoplastic glands, with increased mitosis and nucleocytoplasmic ratio. There is formation of glands, nests and sheets along with necrosis. The white arrow shows the normal splenic tissue and the blue arrow shows the tumor – spleen parenchyma interface.

US, CT and magnetic resonance imaging (MRI) are the preferred diagnostic modalities while histopathology after splenectomy confirms the type of tumor. La Fianza and Madonia retrospectively compared US and CT in detection of splenic metastasis after curative surgery; both provided high accuracy, sensitivity and specificity however the micronodular pattern of splenic involvement gave false positive findings at US [4]. It is the prudent and regular use of these imaging modalities during follow up that has increased the number of case reports reported in the last decade. The diagnosis in our patient was confirmed on histopathology after splenectomy however the space occupying lesion and splenomegaly were detected very accurately initially by US and later by CT. Immunohistochemistry for endometroid adenocarcinoma is also diagnostic; typically these tumors are CK7, CA-125 positive and CK20 negative [5-7]. Our patient had a similar immunohistochemical profile. Splenic metastasis have 3 major macroscopic patterns: macronodular, micronodular and diffuse [8]. The cancer lesions usually affect the upper or lower pole, the hilum of the spleen and rarely infiltrate the capsule [9]. Analysis of the post splenectomy specimen of our patient revealed diffuse extensive infiltration of the parenchyma with involvement of the hilum and lower pole and complete sparing of the capsule as shown in Figures 3 and 5. Capsular metastasis is usually due to disseminated disease and commonly from breast, ovarian or melanoma malignancies [1]. Usually, endometrial carcinoma metastasizes after invasion of the myometrium and periuterine structures to neighboring lymph nodes, liver, lungs, bones and other distal organs; distant metastasis is often independent of the degree of differentiation [10,11]. Splenic metastasis from endometrial carcinoma are usually solitary, located in the splenic parenchyma and spread via the haematogenous route to the spleen. Modes of spread from other malignancies include haematgonous, lymphogenous, direct invasion or abdominal cavity implantation. Solitary disease indicates a more indolent or moderate progress of disease as was the case with our patient [2].

The stage of endometroid endometrial carcinoma in our patient was 1 a and the tumor was moderately well differentiated. The literature as such does not predict any association between time of presentation and initial stage of tumor. The past reports in the literature all report the same histological type of the tumor.

An interesting fact about solitary splenic metastasis from the endometrial adenocarcinoma is that they characteristically present after a period of clinical latency; the literature describes 14 such cases with a range of presentation times for relapse from 11 months to 120 months with a mean time of 34.8 months. Our patient presented after 50 months but started having symptoms at 46 months after surgery. This phenomenon has been postulated to be due to growth of early blood borne disseminated cancer cells within the spleen [1]. Normally, the presence of splenic metastasis in a patient with widespread disseminated disease carries no importance due to their grave prognosis. However, isolated splenic metstasis like in our patient points to a favorable prognosis.

The relative rarity of metastatic involvement of the spleen is speculated to be due to 2 reasons. Mechanical factors including a constant flow of blood through the sharply angulated splenic artery, rhythmic contractions of the splenic capsule and the lack of afferent lymphatics limiting lymphogenous metastasis all impede implantation of blood borne cancer cells. Secondly; the splenic microenvironment itself exerts an inhibitory influence to growth of metastatic cells [3].

The treatment of choice is splenectomy; this not only alleviates the distressing painful splenomegaly like in our patient but it also acts as a precaution against dreadful complications like splenic rupture and splenic vein thrombosis. It also decreases chances of spread of the tumor to distal areas from the spleen and provides the potential for cure or extended survival [12].

After splenectomy our patient was completely pain free; she was referred for chemotherapy and is currently undergoing treatment.

## Conclusion

In conclusion, although metastatic involvement of the spleen from endometrial adenocarcinoma is rare, it is still a potential site for disease asylum. The reported cases are increasing due to advancements in imaging modalities and more vigilant patient follow ups. Since the condition presents after a period of clinical latency after curative surgery for the primary tumor and the patients remain asymptomatic until the tumor increases in size and creates complications, these patients need prolonged follow up with serial imaging to identify recurrence. Once the lesion is diagnosed, splenectomy followed by adjuvant chemotherapy is the treatment of choice.

### Consent

Written informed consent was obtained from the patient for publication of this Case Report and any accompanying images. A copy of the written consent is available for review by the Editor-in-Chief of this journal.

## Abbreviations

CK-7: Cytokeratin 7; CA-125: Cancer antigen 125; EMA: Epithelial Membrane Antigen; CK-20: Cytokeratin 20; CDX-2: Caudal type homeobox 2; CEA: Carcinoembryonic antigen; US: Ultrasonography; CT: Computerized tomography.

## Competing interests

The authors declare that they have no competing interests.

## Authors’ contribution

AA was part of the surgical team that operated upon and looked after the patient and reviewed the manuscript. ZUA drafted the manuscript, completed the literature review and was part of the surgical team that operated upon and took care of the patient. NZ and MAK were the principal surgeons and reviewed the manuscript. TNM and AZM reviewed and revised the manuscript critically, approved its final version for being sent for publication and were part of the consultant team that made pivotal decisions regarding the patient treatment. All authors read and approved the manuscript.
